# Society Meetings

**Published:** 1910-01

**Authors:** 


### SOCIETY MEETINGS.

The Medical Society of the State of New York, will hold its one hundred and fourth annual meeting at Albany, January 25 and 26, 1910, under the presidency of Dr. Charles G. Stockton of Buffalo. The provisional program is as follows:

*Symposium on Appendicitis.*

Under the Auspices of the New York Surgical Society.

Appendicitis in Children, Charles M. Dowd, New York.

Masked Appendicitis, George E. Brewer, New York.

Conditions Simulating Appendicitis, A. B. Johnson, New York.

When to Operate in Appendicitis, Joseph A. Blake, New York.

Deductions to be Made from 1,000 Hospital Cases, Clarence A. McWilliams, New York.

Appendicitis, Roswell Park, Buffalo.

*Symposium on the Heart.*

Elements of Prognosis in Valvular Disease of the Heart, R. Abrahams, New York.

Dilatation of the Heart, Wesley T. Mulligan, Rochester.

*Symposium on Vaccine.*

Vaccine Therapy with the Report of a Case of Human Glanders, A. T. Bristow, Brooklyn.

Vaccine Therapy, Frank Billings, Chicago.

Treatment of Surgical Tuberculosis by Vaccines, James A. MacLeod, Buffalo.

Vaccine Treatment of Surgical Tuberculosis, Lewis L. McArthur, Chicago.

*Symposium on Bone and Joint Changes.*

Tuberculosis of the Bones and Joints, Leonard W. Ely, New York.

Metabolism, Its Relation to Bone and Joint Changes, William H. Porter, New York.

The Joint Changes as Related to the System Diseases of the Cord, Bernard Sachs, New York.

The Rheumatisms, Their Etiology and Pathology, Egbert Le Fevre, New York.

Osteitis Deformans (Paget's Disease), with Report of two cases, Henry L. Elsner, Syracuse.

Lantern Slide Demonstration of *x*-ray Pictures of Osteitis Deformans and Stomach and Intestinal Diseases, Clarence E. Coon, Syracuse.

Spleno-Medullary Leukemia; Its Treatment by Röntgen Therapy, with the Report of a Case, Homer E. Smith, Norwich, and L. A. Van Wagner, Sherburne, N. Y.

The Diagnostic Value of Eosinophilia, Ira S. Wile, New York.

Pellagra, C. H. Lavinder, Passed Assistant Surgeon, United States Public Health Marine Hospital Service.

Experimental Poliomyelitis and its bearing upon Epidemic Poliomyelitis. Simon Flexner and Paul A. Lewis, New York.

Test Meal and Feces Examinations, Some New Methods and Their Clinical Value, Anthony Bassler, New York.

Relation of the Physician to the Hospital Training School, Charles Stover, Amsterdam, N. Y.

The Relationship between the State Board of Regents and Training Schools. Joseph Merzbach, Brooklyn.

The Wasserman Reaction in Leprosy, Howard Fox, New York.

Demonstration of the Wasserman and Allied Reactions by the Hoagland Laboratory. (In a separate room.)

Adequacy of the Present Day Treatment of Syphilis, Tested by the Occurrence of Syphilitic Nervous Diseases, Joseph Collins, New York.

The Treatment of Potts Disease, Brainerd H. Whitbeck, New York.

Phlegmenous Gastritis, Richard W. Westbrook, Brooklyn.

A Contribution to the Studies of Tremors, Marcus Neustaeder, New York.

United States Pharmacopeia, Eli H. Long, Buffalo.

The Effect of Alcohol Observed in the Diseases of the Skin, L. Duncan Bulkley, New York.

Some Remarks on Anemias, Charles O. Bosworth, Rochester.

Lumbar Puncture as a Diagnostic and Therapeutic Agent in General Practice, Nelson G. Russell, Buffalo.

Complications of Typhoid Fever Requiring Surgical Treatment, J. B. Harvie, Troy.

The Importance of Care in Closing the Abdominal Incision, LeRoy Broun, New York.

Chauffeur's Fracture, William S. Thomas, New York.

Shall all Fibroid Tumors of the Uterus be Removed with the Knife? Frank DeWitt Reese, Cortland.

Animal Experimentation, Jacob Gould Schurman, President of Cornell University, Ithaca.

Animal Experimentation, J. R. Day, Chancellor of Syracuse University, Syracuse.

Some Medical Aspects of the Food and Drugs Act, Harvey W. Wiley, Chief of the Bureau of Chemistry, Department of Agriculture, Washington, D. C.

Obituary of Hamilton Dox Wey, Henry L. Elsner, Syracuse.

---

The Medical Society of the County of Erie held its 88th Annual Meeting in the auditorium of the Y. M. C. A. Building, Monday evening, December 20, 1909, under the presidency of Dr. Charles A. Wall. The following named officers and committees were chosen for the year 1910: President, Grover W. Wende; vice-presidents, Bernard Cohen and Frank A. Helwig of Akron; secretary, Franklin C. Gram and treasurer, Albert T. Lytle. The five members of the board of censors chosen are: John H. Grant, Francis E. Fronczak, DeLancey Rochester, Walter D. Greene and George L. Brown. T. H. McKee was elected chairman of the membership committee and Ernest Wende chairman of the public health committee, F. Park Lewis was made committee on legislation. Drs. Arthur W. Hurd, Grover W. Wende, William H. Thornton and Bernard Cohen, were appointed delegates to the Medical Society of the State of New York.

For lack of time the scientific business was postponed until the next meeting. A vote of thanks was tendered to Mayor Adam for his gift of the tuberculosis hospital site and it was recommended that the Police Court be requested to pay more attention to the recommendations of the board of censors in regard to persons practising without a license.

---

American Medical Association—Special conference on Medical Education and Medical Legislation.—A special conference on Medical Education and Legislation will be held at the Congress

Hotel (formerly the Auditorium Annex), Chicago, Monday, Tuesday and Wednesday, February 28, March 1 and 2, 1910, the session to begin at 10 o'clock Monday morning.

On Monday the Council on Medical Education will hold its sixth annual conference. A report will be presented showing the present status of the medical colleges in the United States and another report giving practical tests in state license examinations. Other important topics bearing on medical education will be discussed.

On Tuesday there will be a joint conference on medical education and medical legislation, at which the essentials of a model medical practice act will be considered.

On Wednesday the Committee on Medical Legislation will hold its annual conference, discussing a national bureau of health, vital statistics, pure food and drugs, expert testimony, and other live topics.

Officers and members of state medical licensing boards, officers of state medical associations, members of the national legislative council, university presidents, college professors and others interested in medical education and medical legislation, are most cordially invited to attend this conference and to participate in the discussions.

COUNCIL ON MEDICAL EDUCATION,
*N. P. Colwell, Secretary,*
COMMITTEE ON MEDICAL LEGISLATION,
*Frederick R. Green, Secretary.*

---

THE Medical Society of the County of Chautauqua, held its annual meeting at the Gratiot Hotel, Dunkirk, December 14, 1909. At the morning session, these officers were elected for the ensuing year: president, Edgar Rood of Westfield; first vice-president, E. A. Scofield of Bemus Point; second vice-president, H. A. Eastman of Jamestown; secretary and treasurer, J. W. Morris of Jamestown; censors, V. M. Garfield, of Fredonia, George F. Smith, of Falconer and E. M. Scofield of Jamestown. Several new members were elected. Dinner was served at 1 o'clock and at 2 o'clock the afternoon session began. The annual address was given by Dr. Morris N. Bemus of Jamestown. Papers were read by Lester D. Bowman of Jamestown, V. D. Bozovsky of Dunkirk, V. M. Griswold of Fredonia, and F. H. Nichols of Jamestown.

---

THE Medical Society of the County of Wayne has elected officers as follows: president, Thomas H. Hallett of Clyde; vicepresident, Herman L. Chase of Palmyra; secretary, William J. Bott of Palmyra; treasurer, M. A. Veeder of Lyons; board of censors, George D. York of Newark, Myron E. Carmer of Lyons and George D. Winchell of Rose.

---

The Rochester Academy of Medicine held meetings during the month of December, 1909, at the Hotel Seneca, as follows:

*General Medicine.*—Wednesday evening, December 1. Program: Review and demonstration of Professor Hiss's treatment of acute infections by the use of leukocyte extracts, John R. Williams.

*Surgery.*—Wednesday evening, December 8. Program: Gastric symptoms in certain cases, Williams V. Ewers; Review of modern surgery of the gastrointestinal tract, illustrated by lantern slides, Thomas Jameson.

*Obstetrics.*—Wednesday evening, December 15. Program: Toxins and the liver, William M. Brown.

---

The Buffalo Academy of Medicine held meetings during the month of December, 1909, as follows:

*Section of Surgery.*—Tuesday evening, December 7. Program: Practical suggestions concerning the surgical treatment of certain ano-rectal affections, entroptosis, invagination, and chronic diarrhea, Samuel G. Gant, Professor Rectal and Anal Surgery, New York Post-Graduate Medical School; J. A. Gardner reported a case of Acute fulminating gangrene of the penis and scrotum, with known port of entry, terminating fatally.

*Section of Medicine.*—Monday evening, December 13. Program: Bacteriology of acute infections of the respiratory tract in children, L. Emmett Holt, Professor of Diseases of Children in the College of Physicians and Surgeons (Columbia University), New York. Discussion opened by Charles G. Stockton and Irving M. Snow. Walter S. Goodale presented a case of noma with recovery.

*Section of Obstetrics and Gynecology.*—Tuesday evening, December 21. Program: The aftermath of childbirth, W. P. Manton, Detroit, chairman of obstetrical section of the American Medical Association. Discussion opened by C. C. Frederick.

*Section of Pathology.*—Tuesday evening, December 28. Program: The relation of the internal secretions to surgical conditions, Roswell Park.

